# Supplementary material for: Biochemical, Structural Analysis, and Docking Studies of Spiropyrazoline Derivatives
Source: Int J Mol Sci. 2022 May 27;23(11):6061. doi: 10.3390/ijms23116061 (PMC9181777; doi:10.3390/ijms23116061)
Supplement: Supplementary file 1 [file ijms-23-06061-s001.zip › ijms-1724001-supplementary.pdf]

Biochemical, structural analysis and docking studies of spiropyrazoline derivatives

Angelika A. Adamus-Grabicka [1*], Mateusz Daśko [2], Pawel Hikisz [3] , Joachim Kusz [4], Magdalena Malecka [5] and Elzbieta Budzisz [6*]

[1] Department of Bioinorganic Chemistry, Faculty of Pharmacy, Medical University of Lodz, Muszynskiego 1, Lodz, 90-151, Poland
[2] Department of Inorganic Chemistry, Faculty of Chemistry, Gdansk University of Technology, Narutowicza 11/12, 80-233 Gdansk, Poland
[3] Department of Molecular Biophysics, Faculty of Biology and Environmental Protection, University of Lodz, Pomorska 141/143, 90-236 Lodz, Poland
[4] Institute of Physics, University of Silesia, 75 Pulku Piechoty 1, Chorzow, 41-500, Poland
[5] Department of Physical Chemistry, Faculty of Chemistry, University of Lodz, Pomorska 163/165, Lodz, 90-236, Poland
[6] Department of the Chemistry of Cosmetic Raw Materials, Faculty of Pharmacy, Medical University of Lodz, Muszynskiego 1, Lodz, 90-151, Poland
* Correspondence: elzbieta.budzisz@umed.lodz.pl (E.B.), +48 42 272 55 95; angelika.adamus@umed.lodz.pl (A.A.A-G), +48 42 677 92 26

Diazomethane was prepared for the synthesis spiropyrazolines analogues as follows:

Briefly, 50% aqueous potassium hydroxide solution (30 mL) and 100 mL diethyl ether was placed in a 500 mL flat-bottomed flask. The solution was cooled in an ice-salt mixture to 5 °C and 0.2 M of *N*-nitrosomethylurea (10.3 g) was added in portions. The mixture was stirred. The solution turned yellow and was placed in a previously-cooled separatory funnel. The solution was separated and the ether layer of the diazomethane was placed in a flask covered with pellets of potassium hydroxide. The aqueous layer was placed into a flask with an aqueous solution of benzoic acid.

S1. Synthesis of 5′-(3-methylphenyl)-2-phenyl-4′,5′-dihydro-4*H*-spiro[chromano-3,3′-pirazol]-4-one **(compound I)**

2.5 mmol (0.921 g) of (*E*)-3-(3-methylbenzylidene)-2-phenylchroman-4-one was dissolved in anhydrous acetone (5 mL) to give a light yellow solution. The flask containing the solution was placed in the ice bath and an ethereal solution of diazomethane (10 mmol) was added in excess. The mixture in the flask was left in the freezer for 48 h. The yellow precipitate was filtered off and purified by column chromatography and crystallized from methanol.

Yield: 51.3% **m.p**. 131–132.5 °C. **MS (ESI+)**: m/z 369.4 $C_{24}H_{20}N_2O_2$ [M + H]+. **IR (KBr) *ν*(cm−1)**: 3027 (C-H aromat.), 2975 (C-H aliph.), 1678 (C=O), 1602, 1579, 1472, 1461 (C=C, N=N), 1300 (C-N), 1211 (C-O), 1146 (C-O-C). **^{1}*H*-NMR (DMSO-d$_6$) δ (ppm):** 1.99 (3H, s, CH3), 3.87 (1H, dd, J_{AB} = 3.1Hz, J_{BX} = 6.9 Hz, CH), 4.96 (1H, dd, J_{AB} = 18 Hz, J_{BX} = 7.15 Hz CH_2), 6.19 (1H, s, C2-H), 6.34 (1H, dd, J_{AB} = 18Hz, J_{BX} = 3Hz CH_2), 6.85–7.86 (13H, m, C-H aromat.) **^{13}*C*-NMR (DMSO-d$_6$) δ (ppm):** 20.6 (CH), 39.8 (CH_3), 81.1 (C2-H), 118.6, 121.8, 126.8, 127.8, 128.2, 128.6, 138.0 (CH arom) 102.2, 119.8, 134.7, 134.9, 136.1, 159.4 (C arom), 185.5 (C=O). **Anal. Calc. For $C_{24}H_{20}N_2O_2$** (M = 368.44 g/mol) %C: 78.23; %H: 5.42; %N: 7.59. Found %C: 78.47; %H: 5.35; %N: 7.43.

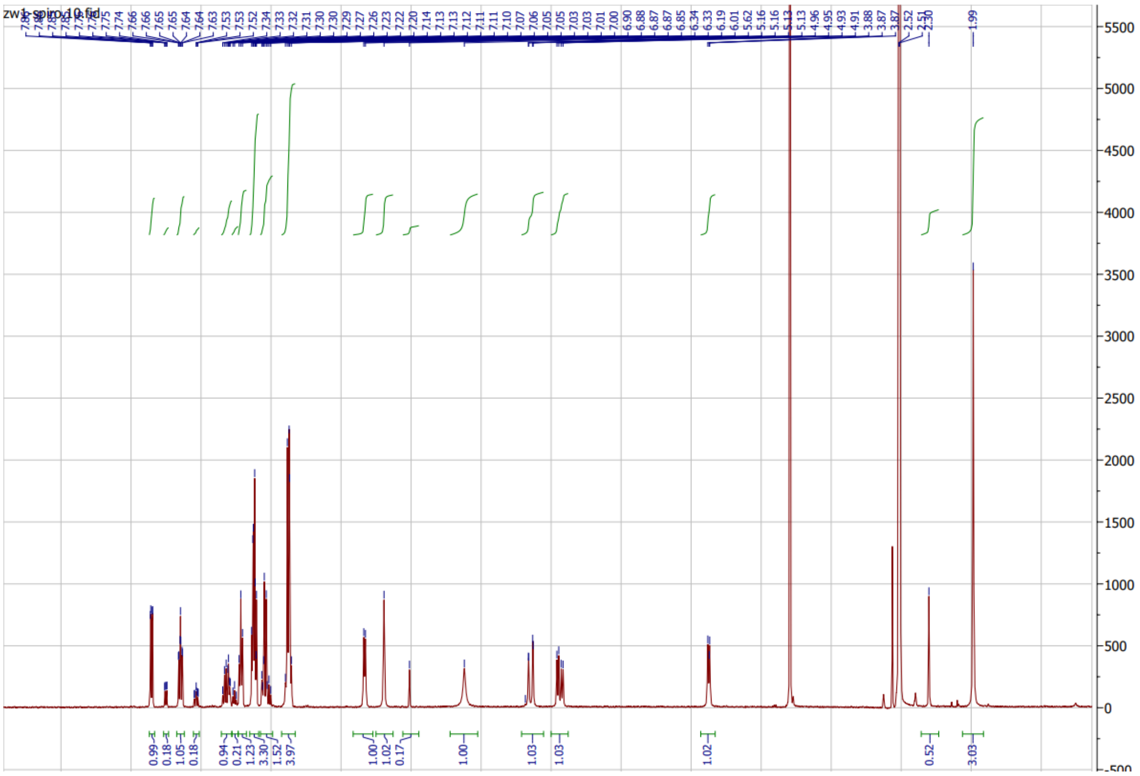

Figure S1a. ^{1}H **NMR spectrum of compound I.**

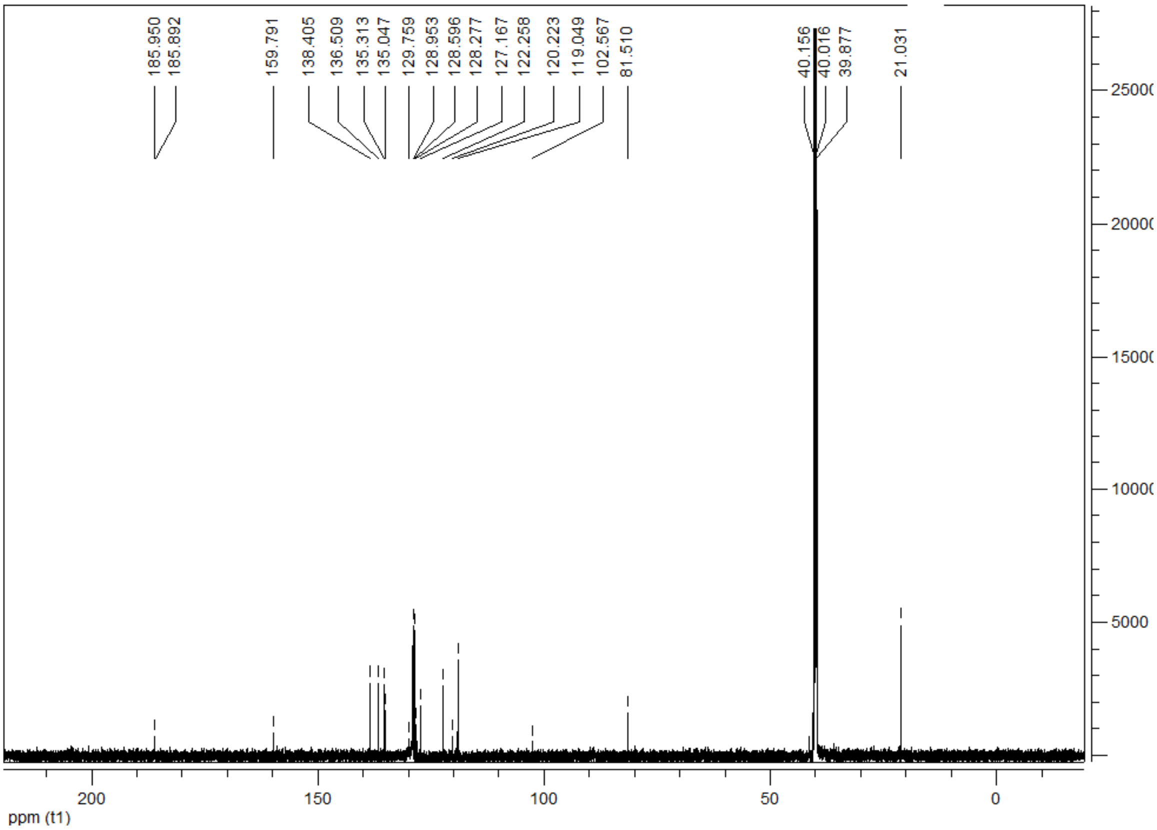

Figure S1b. ^{13}C**NMR spectrum of compound I.**

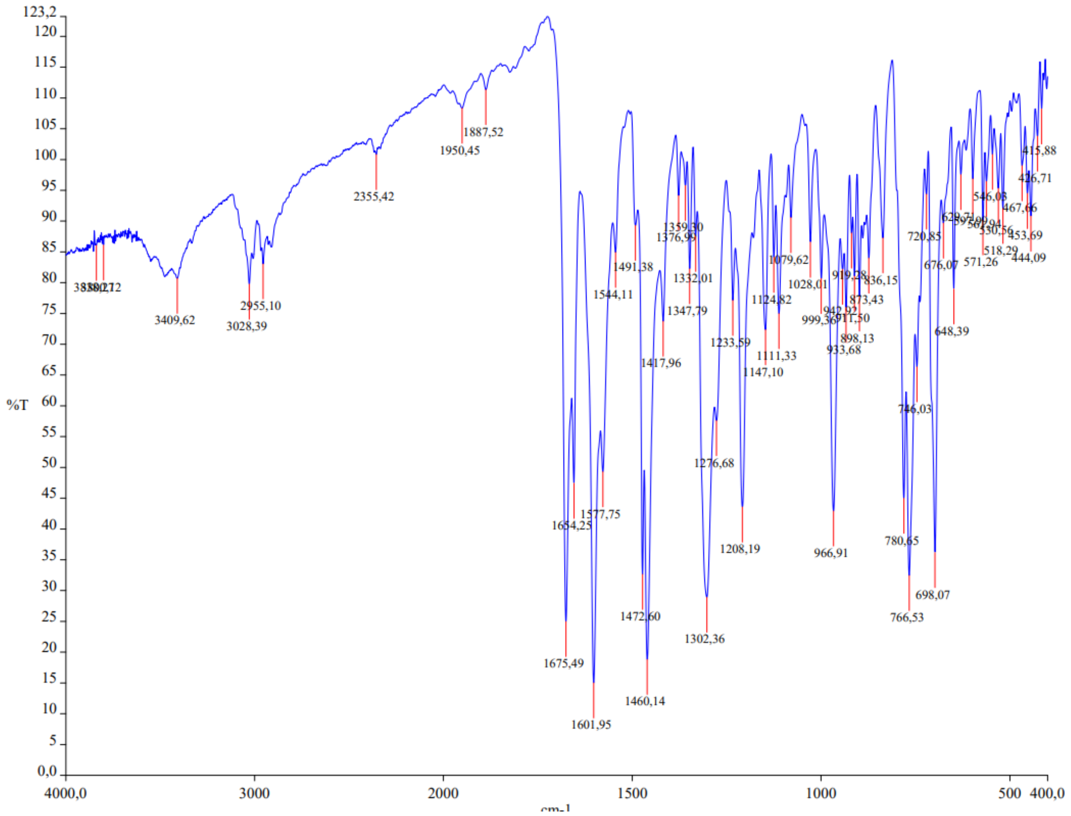

Figure S1c. IR spectrum of compound I.

S2. Synthesis of 5′-(4-methylphenyl)-2-phenyl-4′,5′-dihydro-4*H*-spiro[chromano-3,3′-pirazol]-4-one **(compound II)**

2.5 mmol (0.921 g) of (*E*)-3-(4-methylbenzylidene)-2-phenylchroman-4-one was dissolved in anhydrous acetone (5 mL) to give a light yellow solution. The flask containing the solution was placed in the ice bath and an ethereal solution of diazomethane (10 mmol) was added in excess. The mixture in the flask was left in the freezer for 48 h. The yellow precipitate was filtered off and purified by column chromatography and crystallized from methanol.

Yield: 44% **m.p.** 138.2–140 °C. **MS (ESI+):** m/z 369.5 $C_{24}H_{20}N_2O_2$ [M + H]+. **IR (KBr) *ν*(cm−1):** 3027 (C-H aromat.), 2975, 2928 (C-H aliph.), 1678 (C=O), 1602, 1579 1549, 1514, 1461 (C=C, N=N), 1300 (C-N), 1211 (C-O), 1146 (C-O-C). **^{1}*H*-NMR (DMSO-d$_6$) δ (ppm):** 1.98 (3H, s, CH3), 3.88 (1H, dd, J_{AB} = 7.6 Hz, J_{BX} = 6.9 Hz, CH), 4.95 (1H, dd, J_{AB} = 12.27 Hz, J_{BX} = 1.96 Hz CH_2), 5.13 (1H, dd, J_{AB} = 12.27 Hz, J_{BX} = 7.15 Hz CH_2) 6,19 (1H, s, C2-H), 6.87–7.86 (13H, m, C-H aromat). **^{13}*C*-NMR ($CDCl_3$) δ (ppm):** 23.6 (CH_3), 55,2 (CH), 77.3 (CH), 79.5 (C2-H), 112.3, 113.9, 118.2, 119.1, 123.4, 127.3, 128.5, 129.8, 138.9 (CH arom), 102.6, 121.3, 135.7, 142.6, 152.8, 158.7, 159.6 (C arom), 185.3 (C=O). **Anal. Calc. For $C_{24}H_{20}N_2O_2$** (M = 368.44 g/mol) %C: 78.23; %H: 5.42; %N: 7.59. Found %C: 78.37; %H: 5.21; %N: 7.49.

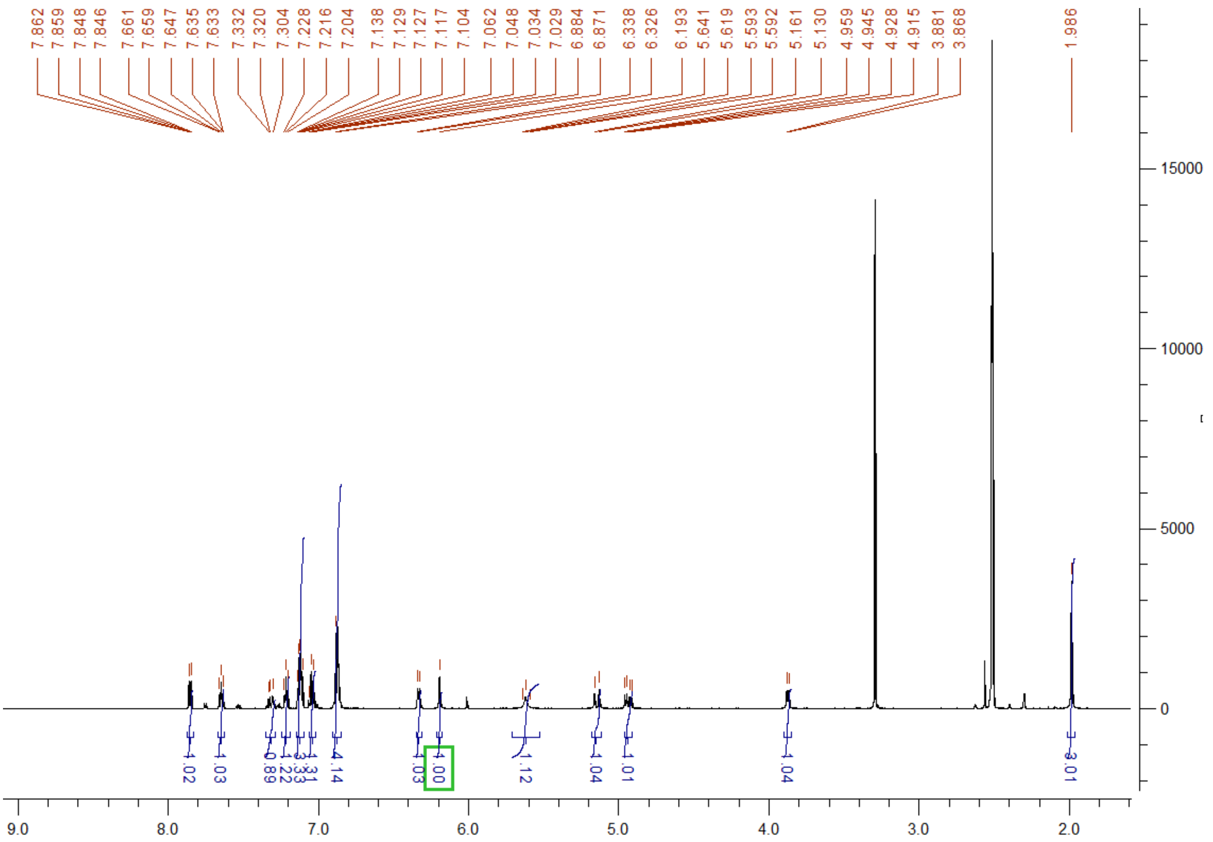

Figure S2a. [1]*H* **NMR spectrum of compound II.**

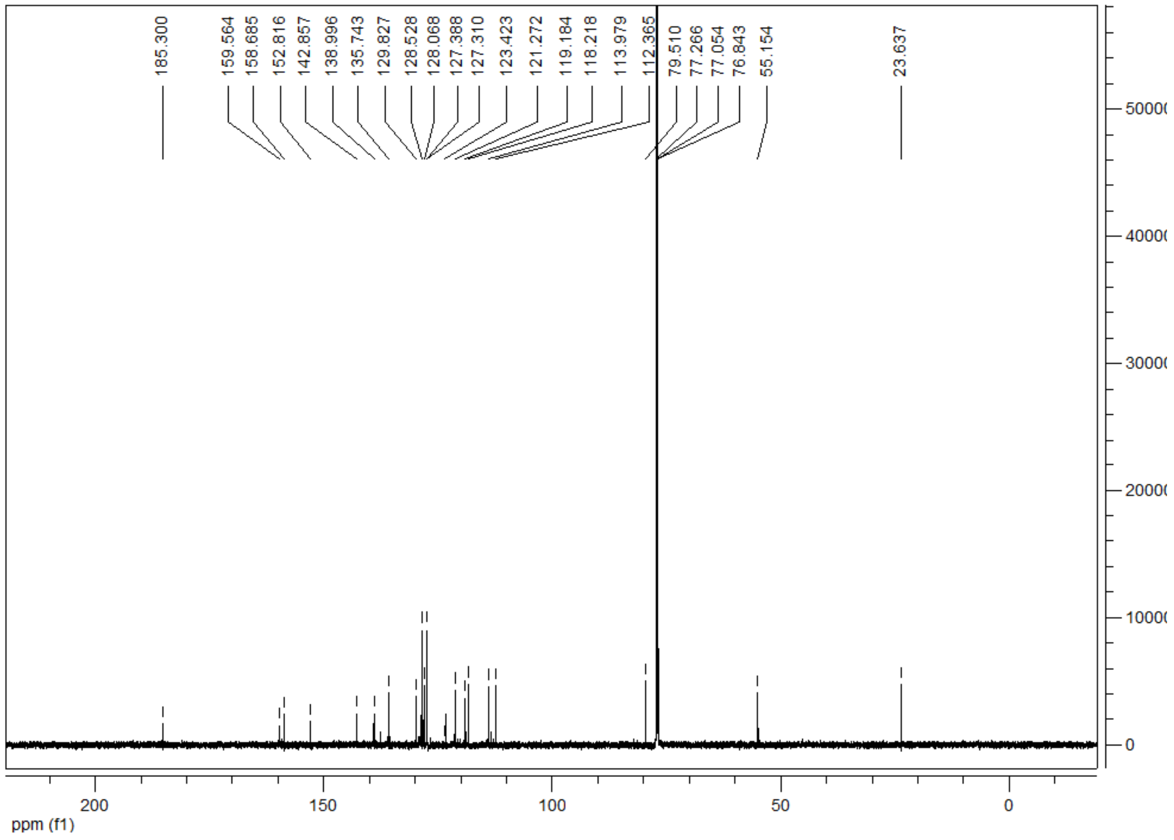

Figure S2b. [13]*C***NMR spectrum of compound II.**

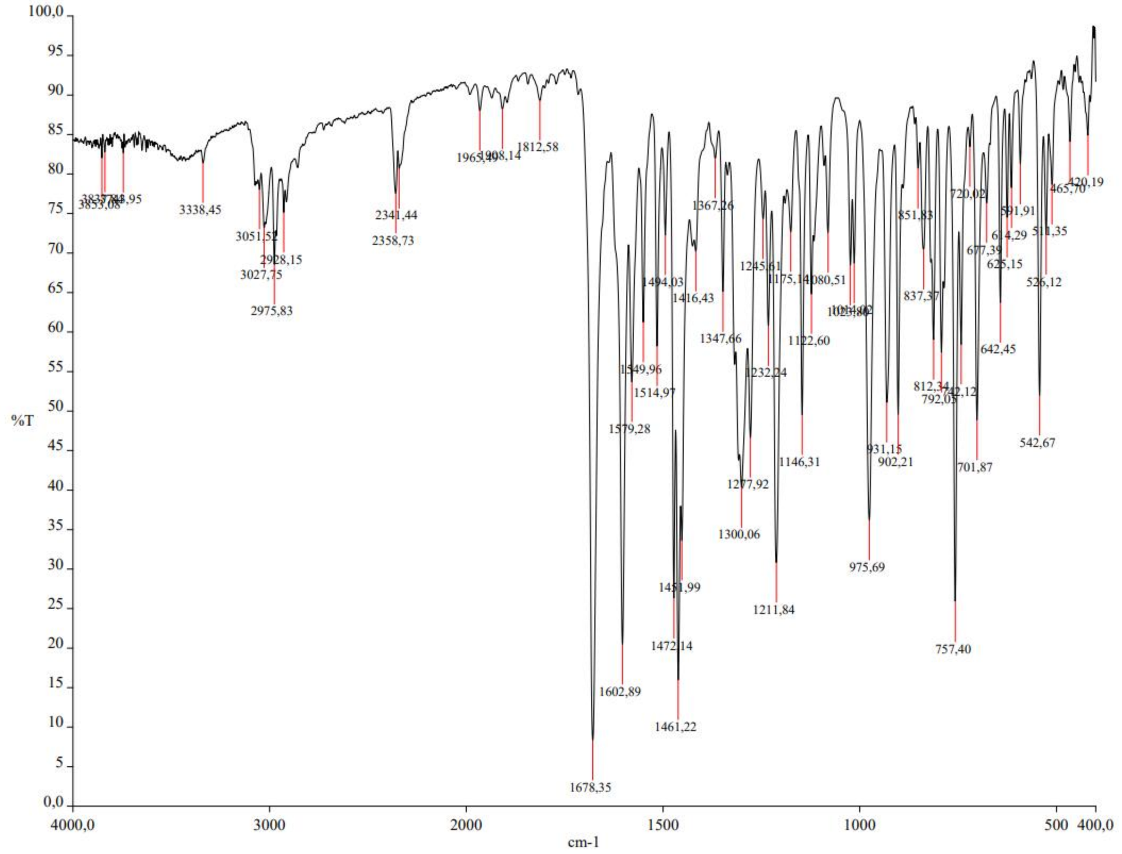

Figure S2c. IR spectrum of compound II.
